# Uptake of Voluntary Counselling and Testing among Young People Participating in an HIV Prevention Trial: Comparison of Opt-Out and Opt-In Strategies

**DOI:** 10.1371/journal.pone.0042108

**Published:** 2012-07-25

**Authors:** Kathy Baisley, Aoife M. Doyle, John Changalucha, Kaballa Maganja, Deborah Watson-Jones, Richard Hayes, David Ross

**Affiliations:** 1 London School of Hygiene and Tropical Medicine, London, United Kingdom; 2 Mwanza Intervention Trials Unit, National Institute for Medical Research, Mwanza, Tanzania; 3 National Institute for Medical Research, Mwanza, Tanzania; Africa Centre for Health and Population Studies - University of KwaZulu-Natal, South Africa

## Abstract

**Background:**

HIV voluntary counselling and testing (VCT) is an integral component of HIV prevention and treatment programmes. However, testing coverage in sub-Saharan Africa is still low, particularly among young people. As treatment becomes more widely available, strategies to expand VCT coverage are critically important. We compare VCT uptake using two delivery strategies (opt-in and opt-out) within the MEMA kwa Vijana trial in 20 communities in northwest Tanzania.

**Methods:**

We analysed data from 12,590 young persons (median (IQR) age 22 years (20–23)) to assess the effect of delivery strategy on VCT uptake. Ten communities used an opt-in approach and 10 used opt-out, balanced across intervention and control. Conditional logistic regression was used to examine factors associated with uptake within each strategy.

**Results:**

VCT uptake was significantly higher with the opt-out approach (90.9% vs 60.5%, prevalence ratio = 1.51, CI = 1.41–1.62). Among females, uptake in the opt-out approach was associated with decreased knowledge of HIV acquisition, sex with a casual partner, and being HSV-2 seronegative; among males, uptake was associated with lower education and increasing lifetime partners. In contrast, uptake using the opt-in approach varied by ethnic group, religion and marital status, and increased with increasing knowledge of STI acquisition (males) or pregnancy prevention (females).

**Conclusion:**

VCT uptake among young people was extremely high when offered an opt-out strategy. Sociodemographic and knowledge factors affected uptake in different ways depending on delivery strategy. Increased knowledge may increase young persons' self-efficacy, which may have a different impact on testing uptake, depending on the approach used.

## Introduction

As access to antiretroviral therapy (ART) becomes more widely available for the treatment of HIV infection, expanding access to, and use of, VCT is critically important.

‘Client-initiated’ VCT, where individuals proactively seek HIV testing, remains the primary VCT model in many sub-Saharan African countries. Its availability is constrained by a shortage of skilled service providers and weak health infrastructure. Barriers to uptake of client-initiated VCT include personal perceptions of risk, negative perceptions of health services or of HIV testing services, and fear of stigma and discrimination [1], [2], [3].

‘Provider-initiated’ testing and counselling (PITC) is an alternative approach to VCT, where individuals are informed that they will receive an HIV test as part of general medical screening or clinical management unless they opt out. This model has been promoted by WHO and UNAIDS to increase opportunities for HIV diagnosis, and by 2009, two-thirds of countries in sub-Saharan Africa had introduced policies supporting PITC [4].

Repeat population-based surveys in 9 African countries in 2003–2009 have shown an increase in VCT uptake in recent years [4]. In Tanzania, an estimated 30% of women and 25% of men aged 15–49 in 2009–2010 had been tested for HIV in the past 12 months, compared with 6% and 7%, respectively, in 2004–2005 [5]. However, in many countries in sub-Saharan Africa, VCT uptake is lower among young people than in older age groups, and the difference by age is greater among men than women [6],[7].

Between 1998–2008, a community-randomised controlled trial was conducted in rural Tanzania to evaluate the impact of the MEMA kwa Vijana (“Good things for young people”) adolescent sexual and reproductive health intervention. The trial was conducted in 20 communities, with 10 randomly chosen to receive the intervention. Impact evaluations conducted 3 years (2001/2) and 9 years (2007/8) after the start of the intervention demonstrated that it had led to an improvement in young people's sexual and reproductive health knowledge and reported attitudes and in some reported sexual behaviours [8],[9]. However, no significant impact was seen on the prevalence of HIV, other STIs or pregnancies.

The trial intervention did not specifically aim to increase VCT uptake and VCT uptake was not a pre-specified trial outcome. However, in the 2007/8 follow-up survey, we compared VCT uptake using an opt-in versus an opt-out approach, and examined factors associated with uptake under each strategy. Since this was not a main aim of the trial, communities were not randomised to VCT delivery strategy, but were balanced in terms of intervention and control arms within each VCT strategy.

## Methods

### Ethics

The trial protocol received ethical and research clearance from the Tanzanian Medical Research Coordinating Committee and the Ethics Committee of the London School of Hygiene and Tropical Medicine. Signed informed consent was obtained from each participant on the day of the survey. Additional written consent was obtained from parents for participants under the age of 18 years.

### Study design

The overall MEMA kwa Vijana intervention and study design have been described previously [10], [11]. In 2007–2008, a cross-sectional survey was conducted to evaluate the long-term impact of the MEMA kwa Vijana intervention [9].

A household census in the 20 trial communities identified potentially eligible young people who were invited to a survey at a central location 2–3 days later. Young people who had attended one or more of the last 3 years of primary school (standard 5, 6 or 7) in a school within a trial community between 1999–2002 inclusive were eligible for the survey.

Eligible individuals who gave informed consent were interviewed on sexual knowledge, attitudes and behaviour in a 20–30 minute structured questionnaire administered by a same-sex, 21–29 year-old research assistant. Laboratory samples (serum and urine) were collected by trained technicians, and a clinician checked for symptoms (males and females) and clinical signs (males only) suggestive of STI.

The majority (91%) of participants were surveyed from June 2007–May 2008. However, in order to include additional eligible young people, all trial communities, nearby secondary schools, and major migration points within the Lake Zone of Tanzania were revisited in June–July 2008. The survey teams for the re-visit phase were different from those in the main phase, and used the opt-in VCT delivery strategy only. For consistency, the analysis of VCT uptake presented here includes only those participants seen by the two trial survey teams during the main survey and excludes participants who attended during the re-visit phase.

### HIV counselling and testing

The interviews and clinical procedures were conducted by two survey teams, with each team visiting 10 communities. In both survey teams, VCT was offered to all participants as the final step of the study visit, immediately after the interview and clinical assessment were completed. Team 1 offered VCT using an opt-out approach, whereby all participants saw a counsellor who offered them HIV counselling and testing. Team 2 offered VCT using an opt-in approach, whereby the main study interviewer told all participants that they could visit a counselling room and see an on-site counsellor in order to have an HIV test if they desired. Since evaluation of effects on VCT uptake was not a primary aim of the trial, communities were not randomised to VCT delivery strategy. However, each survey team was allocated to 5 intervention and 5 control communities, so VCT delivery was balanced in terms of the intervention and control arms. For logistical reasons, communities that were in the same geographical area were visited by the same team.

Each team had one trained male and one trained female counsellor. VCT sessions were held in a separate, private room, at the same location where the other study procedures were conducted. Participants were informed about the service at the start of the study visit.

Participants received one-to-one pre-test counselling; written consent was obtained before testing. Blood was tested using two parallel HIV rapid tests (SD Bioline HIV-1/2 3.0 (Standard Diagnostics Inc) and Determine HIV1/2 (Abbott Laboratories)). All participants received post-test counselling. Those who tested positive were referred to the nearest health facility offering ART so that their eligibility for treatment could be assessed, and were provided with a referral letter and enough money to cover their transport costs.

### Questionnaire

As part of the main interview, participants were asked three questions on each of three knowledge domains (HIV acquisition, STI acquisition and pregnancy prevention), and three questions related to their sexual attitudes [9]. A score was constructed for each domain, ranging from 0 (no correct answers) to 3 (all answers correct).

Participants were also asked about lifetime sexual behaviour, sexual behaviours in the past 12 months, including detailed information about the last three partners, contraception and pregnancy.

### Laboratory methods

The laboratory methods have been described elsewhere [9]. Briefly, sera were tested by HIV ELISA (Murex HIV 1.2.0, Murex Biotech, UK; Vironostika HIV UniformII plusO, bioMérieux, the Netherlands). Discordant or indeterminate samples were retested. If results were not resolved on retesting, the sample was tested by HIV-1 p24 Ag EIA (Biorad Genetic Systems, USA). P24 negative samples were tested with a line immunoassay (INNO-LIA™ HIV-I/II, Innogenetics, Belgium). Sera were tested for antibodies to HSV2 (Kalon HSV Type2 IgG ELISA, Kalon Biologicals, Guildford, UK). Syphilis was tested using the *Treponema pallidum* particle agglutination (TPPA) test (Serodia, Fujirebio, Japan). Those positive on TPPA were tested for active syphilis using the rapid plasma reagin (RPR) test (Immutrep, Omega Diagnostics, Hillfoot, UK).

### Statistical analysis

Data were double-entered and verified, and were analysed using Stata 11. Participant characteristics were tabulated by study team and compared using the Pearson chi-squared statistic with the second-order correction of Rao and Scott to account for the clustered design

We examined the effect of delivery strategy on the prevalence of VCT uptake, using methods for cluster randomised trials [12]. Uptake was measured using prevalence ratios (PR), calculated as the ratio of geometric mean prevalence of VCT uptake for the 10 communities in each strategy. The 95% confidence interval (CI) was calculated using the residual mean square from a two-way analysis of variance (ANOVA) of log prevalence on stratum and strategy. Since communities were not randomised to VCT delivery strategy (although they were balanced by intervention and control), analyses were also adjusted for stratum, age group, sex, ethnic group and education level. Adjusted prevalence ratios (aPR) were calculated as the geometric mean ratio of observed to expected events, with logistic regression used to estimate the expected number of events, adjusted for individual-level covariates.

Next we examined the effect of the MEMA kwa Vijana intervention on overall VCT uptake, using the same method. In addition, we did separate analyses within each survey team, to evaluate the impact of the MEMA intervention on VCT uptake within each delivery strategy.

Lastly, we investigated factors associated with VCT uptake separately within each survey team. Since some of the associations might have differed between men and women, all analyses were stratified by sex. We used conditional logistic regression to estimate odds ratios (OR) and 95% CI, conditioning on community to account for within-community correlation. Potential determinants of VCT uptake were examined using a conceptual framework with four levels: sociodemographic factors, sexual knowledge and attitudes, behavioural factors, and biological factors. First, sociodemographic factors that were associated with VCT uptake at p<0.10 were included in a multivariable model; those remaining independently associated at p<0.10 were retained in a core model. Sexual knowledge and attitude factors were added to this core model one by one. Those that were associated with VCT uptake at p<0.10, after adjusting for sociodemographic factors, were included in a multivariable model and retained if they remained significant at p<0.10. Associations of VCT uptake with behavioural and then biological factors were determined in a similar way. The final model excluded factors one at a time until all remaining factors were significant at p<0.05.

## Results

### Survey participants

In total 12,590 young persons (5628 females and 6962 males) were surveyed from June 2007–May 2008 and were offered VCT. Overall HIV prevalence was 2.9% (4.0% in females and 1.8% in males). Team 1 (opt-out strategy) surveyed fewer participants than Team 2 (5938 vs 6652, respectively), a higher proportion of males (57% vs 54%, p = 0.02; Table 1), and lower proportion of Sukuma ethnic group (72% vs 87%, p = 0.10). The median (IQR) age of participants surveyed by each team was 22 (20–24) years. The proportion with secondary education or above was lower in the communities visited by Team 1 (10% vs 21%, p<0.001). Otherwise the participants seen by each team were reasonably similar.

**Table 1:** Characteristics of study participants, by VCT strategy.

	Opt-out strategy (Team 1, N = 5938) N (%)	Opt-in strategy (Team 2, N = 6652) N (%)	p-value^1^
**Sex**			
Female	2541 (42.8%)	3087 (46.4%)	0.02
Male	3397 (57.2%)	3565 (53.6%)	
**Age (years)**			
<21	2001 (33.7%)	2200 (33.1%)	0.43
21–22	1628 (27.4%)	1864 (28.0%)	
23–24	1429 (24.1%)	1719 (25.8%)	
≥25	877 (14.8%)	869 (13.1%)	
**Currently married**			
Yes	2784 (46.9%)	2833 (42.6%)	0.18
**Tribe**			
Sukuma	4290 (72.4%)	5779 (87.0%)	0.10
Non-Sukuma	1639 (27.6%)	867 (13.0%)	
**Religion**			
Christian	4854 (81.9%)	5704 (85.9%)	0.39
Muslim	326 (5.5%)	214 (3.2%)	
None/traditional/other	744 (12.6%)	724 (10.9%)	
**Circumcised (males only)**			
Yes	1390 (42.0%)	1363 (38.5%)	
**Highest level of education**			
Secondary or higher	618 (10.4%)	1426 (21.5%)	<0.001
Main occupation			
Business	825 (14.0%)	1230 (18.5%)	0.22
Fishing/farming	3572 (60.5%)	3892 (58.7%)	
Student	1076 (18.2%)	1024 (15.4%)	
None/other	432 (7.3%)	490 (7.4%)	
**Type of site/community**			
Farming/mainly farming	4700 (79.2%)	4955 (74.5%)	0.21
Fishing/mainly fishing	363 (6.1%)	513 (7.7%)	
Trading centre	490 (8.3%)	1164 (17.5%)	
Mining	385 (6.5%)	19 (0.3%)	
**HIV serostatus**			
Positive	144 (2.4%)	190 (2.9%)	0.32

1 Rao-Scott *F* (second-order correction to the Pearson χ^2^ statistic to account for clustered design).

### Impact of strategy on VCT uptake

Overall, 9420 (74.8%) participants accepted VCT. VCT uptake was significantly higher among participants offered the opt-out strategy than those offered opt-in (90.9 vs 60.4%, p<0.001; PR = 1.51, CI = 1.41–1.62; Table 2). Uptake was higher with the opt-out strategy in all age, sex and sociodemographic categories. After adjusting for age, sex, ethnic group and education, participants offered the opt-out strategy were much more likely to accept testing than those offered the opt-in approach (aPR = 1.46, CI = 1.36–1.56).

**Table 2:** Impact of MEMA kwa Vijana intervention and delivery strategy on VCT uptake in males & females.

	EFFECT OF MEMA KWA VIJANA INTERVENTION			
	Intervention n/N (%)	Comparison n/N (%)	Unadjusted PR (95% CI)	Adjusted PR^1^ (95% CI)
**ALL PARTICIPANTS**				
VCT uptake	4873/6489 (75.1)	4547/6101 (74.5)	1.00 (0.78, 1.27)	0.99 (0.78, 1.25)
VCT uptake in Team 1	2822/3128 (90.2)	2577/2810 (91.7)	0.98 (0.94, 1.03)	0.98 (0.93, 1.03)
VCT uptake in Team 2	2051/3361 (61.0)	1970/3291 (59.9)	1.02 (0.85, 1.21)	1.02 (0.86, 1.22)
**MALES**				
VCT uptake	2769/3630 (76.3)	2496/3332 (74.9)	1.01 (0.80, 1.29)	1.01 (0.80, 1.28)
VCT uptake in Team 1	1623/1790 (90.7)	1468/1607 (91.4)	0.99 (0.95, 1.03)	0.99 (0.94, 1.03)
VCT uptake in Team 2	1146/1840 (62.3)	1028/1725 (59.6)	1.04 (0.84, 1.29)	1.04 (0.84, 1.30)
**FEMALES**				
VCT uptake	2104/2859 (73.6)	2051/2769 (74.1)	0.98 (0.77, 1.25)	0.97 (0.76, 1.23)
VCT uptake in Team 1	1199/1338 (89.6)	1109/1203 (92.2)	0.97 (0.92, 1.03)	0.98 (0.92, 1.04)
VCT uptake in Team 2	905/1521 (59.5)	942/1566 (60.2)	0.98 (0.86, 1.13)	0.99 (0.86, 1.14)

	EFFECT OF DELIVERY STRATEGY			
	Opt-out strategy n/N (%)	Opt-in strategy n/N (%)	Unadjusted PR (95% CI)	Adjusted PR^2^ (95% CI)
**ALL PARTICIPANTS**				
VCT uptake	5399/5938 (90.9)	4021/6652 (60.4)	1.51 (1.41, 1.62)	1.46 (1.36, 1.56)
**MALES**				
VCT uptake	3091/3397 (91.0)	2174/3565 (61.0)	1.50 (1.38, 1.62)	1.42 (1.31, 1.53)
**FEMALES**				
VCT uptake	2308/2541 (90.8)	1847/3087 (59.8)	1.53 (1.42, 1.64)	1.50 (1.39, 1.62)

1 Adjusted for sex (analysis in all participants), and stratum, age and tribe.

2 Adjusted for sex (analysis in all participants), and stratum, age, tribe and education.

Within each strategy, there was no evidence of a difference in the proportion of males and females accepting VCT (91.0% vs 90.8%, respectively, using the opt-out strategy, p = 0.80; and 61.0% vs 59.8%, respectively, using the opt-in strategy, p = 0.53).

### Impact of intervention on VCT uptake

There was no evidence of an impact of the MEMA kwa Vijana intervention on VCT uptake overall, in either males or females (PR = 1.01, CI = 0.80–1.29 and PR = 0.98, CI = 0.77–1.25, respectively; Table 2). Furthermore, there was no evidence of an impact of the intervention on VCT uptake with either strategy, or after adjusting for individual-level covariates (Table 2).

### Factors associated with VCT uptake in the opt-out strategy (Team 1)

In females, in the unadjusted analysis, factors associated (p<0.10) with VCT uptake using the opt-out strategy were knowledge of HIV acquisition, sex with a casual partner in the past 12 months and being HSV-2 seronegative (Table 3). In the multivariable analysis, only negative HSV2 serostatus remained independently associated with VCT uptake at p<0.05 (aOR = 0.74, CI = 0.56–0.98; Table 4). There was some evidence that VCT uptake was higher among those reporting a casual partner in the past 12 months (aOR = 1.52, CI = 0.96–2.38), and was inversely associated with knowledge of HIV acquisition (aOR = 0.77, CI = 0.57–1.05 comparing 3 correct vs 0–2 correct answers).

**Table 3:** Factors associated with acceptance of VCT within each strategy.

	OPT-OUT STRATEGY (TEAM 1)				OPT-IN STRATEGY (TEAM 2)			
	Females (N = 2541)		Males (N = 3397)		Females (N = 3087)		Males (N = 3565)	
	number accepting (%)	Unadjusted OR (95% CI)	number accepting (%)	Unadjusted OR (95% CI)	number accepting (%)	Unadjusted OR (95% CI)	number accepting (%)	Unadjusted OR (95% CI)
**SOCIO-DEMOGRAPHIC FACTORS**								
**Age (years)**		P = 0.18		P = 0.74		P = 0.20		P = 0.15
<21	971 (90.9)	1	848 (90.9)	1	712 (60.0)	1	602 (59.4)	1
21–22	647 (89.0)	0.79 [0.58, 1.09]	813 (90.3)	0.92 [0.67, 1.26]	521 (58.6)	0.94 [0.79, 1.12]	585 (60.0)	1.03 [0.86, 1.23]
23–24	504 (92.3)	1.15 [0.79, 1.69]	809 (91.6)	1.09 [0.79, 1.52]	482 (62.4)	1.11 [0.92, 1.34]	584 (61.7)	1.10 [0.92, 1.32]
≥25	184 (92.9)	1.25 [0.69, 2.25]	620 (91.3)	1.06 [0.74, 1.51]	132 (55.0)	0.83 [0.63, 1.11]	403 (64.1)	1.25 [1.02, 1.54]
**Currently married**		P = 0.33		P = 0.55		P = 0.39		P = 0.02
No	963 (91.0)	1	1910 (91.1)	1	809 (60.6)	1	1486 (59.8)	1
Yes	1345 (90.7)	0.87 [0.66, 1.15]	1181 (90.8)	0.93 [0.73, 1.18]	1038 (59.2)	0.94 [0.81, 1.09]	688 (63.7)	1.20 [1.03, 1.39]
**Tribe**		P = 0.78		P = 0.52		P = 0.05		P = 0.50
Sukuma	1737 (91.2)	1	2163 (90.7)	1	1598 (59.3)	1	1893 (61.4)	1
Non-Sukuma	567 (89.7)	0.95 [0.69, 1.32]	924 (91.8)	1.10 [0.82, 1.48]	249 (64.0)	1.28 [1.00, 1.64]	279 (58.4)	0.92 [0.74, 1.16]
**Religion**		P = 0.26		P = 0.79		P = 0.002		P<0.001
Christian	1990 (90.6)	1	2421 (91.1)	1	1687 (60.9)	1	1819 (60.9)	1
Muslim	122 (88.4)	0.83 [0.48, 1.45]	171 (91.0)	0.91 [0.54, 1.54]	53 (54.1)	0.81 [0.54, 1.22]	59 (54.1)	0.63 [0.43, 0.91]
None/other	189 (94.5)	1.57 [0.83, 2.95]	493 (90.6)	0.90 [0.65, 1.24]	103 (48.1)	0.59 [0.44, 0.79]	252 (55.4)	0.70 [0.57, 0.87]
**Circumcised (males)**				P = 0.91				P = 0.005
Yes			1261 (90.7)	1			782 (57.4)	1
No			1754 (91.3)	0.99 [0.76, 1.28]			1381 (63.4)	1.25 [1.07, 1.46]
**Education**		P = 0.31		P = 0.02		P = 0.23		P = 0.10
Secondary or higher	246 (89.1)	1	296 (86.6)	1	223 (59.6)	1	615 (58.5)	1
Primary	1976 (90.9)	1.18 [0.78, 1.79]	2737 (91.5)	1.62 [1.16, 2.28]	1535 (60.3)	0.98 [0.78, 1.23]	1558 (62.6)	1.12 [0.96, 1.31]
Incomplete primary	82 (94.3)	2.03 [0.76, 5.44]	54 (93.1)	1.97 [0.68, 5.70]	87 (53.7)	0.74 [0.51, 1.08]	108 (55.7)	0.85 [0.62, 1.16]
**SEXUAL KNOWLEDGE FACTORS**								
**Knowledge of HIV acquisition**		P = 0.09		P = 0.59		P = 0.98		P = 0.02
0–2 correct	858 (92.7)	1	944 (91.6)	1	660 (60.2)	1	647 (59.1)	1
3 correct	1444 (89.8)	0.77 [0.57, 1.04]	2137 (90.7)	0.93 [0.71, 1.21]	1179 (59.6)	1.00 [0.86, 1.17]	1524 (61.9)	1.20 [1.03, 1.39]
**Knowledge of STI acquisition**		P = 0.75		P = 0.11		P = 0.81		P = 0.007
0–2 correct	1575 (91.2)	1	1509 (92.0)	1	1208 (60.2)	1	1088 (59.4)	1
3 correct	729 (89.9)	0.95 [0.71, 1.27]	1574 (90.1)	0.82 [0.65, 1.05]	633 (59.4)	0.98 [0.84, 1.14]	1081 (62.8)	1.21 [1.05, 1.39]
**Knowledge of pregnancy prevention**		P = 0.50		P = 0.52		P<0.001		P = 0.10
0–2 correct	865 (90.9)	1	731 (91.7)	1	564 (56.1)	1	524 (59.3)	1
3 correct	1438 (90.8)	1.10 [0.83, 1.46]	2349 (90.8)	0.91 [0.68, 1.22]	1277 (61.7)	1.32 [1.13, 1.54]	1644 (61.5)	1.15 [0.98, 1.35]
**Sexual attitudes**		P = 0.45		P = 0.76		P = 0.60		P = 0.58
0–2 correct	2068 (90.7)	1	2181 (91.1)	1	1648 (59.7)	1	1764 (61.4)	1
3 correct	236 (91.5)	1.19 [0.75, 1.91]	906 (90.8)	0.96 [0.74, 1.25]	198 (61.9)	1.07 [0.84, 1.36]	409 (59.4)	0.95 [0.80, 1.13]
**BEHAVIOURAL FACTORS**								
**Lifetime number partners**		P = 0.56		P = 0.02		P = 0.28		P = 0.02
0	179 (92.8)	1.47 [0.81, 2.68]	292 (90.4)	0.98 [0.59, 1.62]	102 (62.2)	1.14 [0.81, 1.62]	161 (54.2)	1
1	720 (90.3)	1	357 (90.6)	1	466 (58.6)	1	210 (57.4)	1.13 [0.83, 1.54]
2 (F) or 2–4 (M)	659 (90.5)	1.02 [0.72, 1.44]	1231 (89.5)	0.87 [0.59, 1.27]	551 (58.1)	0.97 [0.80, 1.18]	819 (60.9)	1.29 [1.00, 1.67]
≥3 (F) or ≥5 (M)	742 (91.2)	1.13 [0.80, 1.59]	1204 (92.9)	1.35 [0.90, 2.02]	725 (61.9)	1.13 [0.94, 1.37]	971 (63.4)	1.44 [1.11, 1.85]
**Pregnancy in primary school?**		P = 0.40		P = 0.13		P = 0.81		P = 0.05
No	2225 (90.7)	1	2975 (90.9)	1	1793 (59.9)	1	2091 (60.7)	1
Yes	83 (93.3)	1.41 [0.61, 3.28]	116 (94.3)	1.74 [0.80, 3.78]	54 (58.7)	0.95 [0.62, 1.45]	83 (70.3)	1.49 [1.00, 2.23]
**Slept away in past 12 m**		P = 0.83		P = 0.87		P = 0.29		P<0.001
No	1172 (91.3)	1	1032 (90.8)	1	979 (59.0)	1	626 (56.5)	1
Yes	1129 (90.3)	0.97 [0.74, 1.28]	2055 (91.1)	1.02 [0.80, 1.31]	867 (60.9)	1.08 [0.93, 1.26]	1546 (63.1)	1.31 [1.13, 1.52]
**Concurrency in past 12 m** 1		P = 0.49		P = 0.07		P = 0.34		P = 0.002
No	2142 (90.8)	1	2170 (90.4)	1	1719 (59.6)	1	1416 (58.9)	1
Yes	166 (91.7)	1.21 [0.69, 2.10]	921 (92.4)	1.28 [0.98, 1.68]	128 (63.7)	1.15 [0.86, 1.56]	758 (65.2)	1.27 [1.09, 1.47]
**Casual partner in past 12 m** 1		P = 0.05		P = 0.05		P = 0.21		P = 0.003
No	1981 (90.5)	1	1714 (90.2)	1	1581 (59.3)	1	931 (57.9)	1
Yes	327 (93.2)	1.53 [0.98, 2.37]	1377 (92.1)	1.27 [1.00, 1.62]	266 (63.2)	1.14 [0.92, 1.42]	1243 (63.6)	1.23 [1.08, 1.41]
**BIOLOGICAL FACTORS**								
**HIV serostatus**		P = 0.30		P = 0.54		P = 0.64		P = 0.22
Negative	2231 (91.3)	1	3018 (91.3)	1	1768 (60.0)	1	2136 (61.2)	1
Positive	62 (87.3)	0.67 [0.33, 1.38]	68 (93.1)	1.32 [0.53, 3.31]	78 (57.4)	0.92 [0.65, 1.31]	37 (68.5)	1.43 [0.80, 2.55]
**HSV-2**		P = 0.06		P = 0.58		P = 0.50		P = 0.17
Negative	1373 (92.1)	1	2258 (91.5)	1	1057 (59.6)	1	1614 (60.9)	1
Positive	920 (89.9)	0.76 [0.58, 1.01]	828 (91.0)	0.93 [0.71, 1.21]	789 (60.2)	1.05 [0.91, 1.22]	559 (62.6)	1.12 [0.95, 1.31]
**Syphilis** 2		P = 0.46		P = 0.81		P = 0.01		P = 0.08
Negative	2137 (91.2)	1	2898 (91.4)	1	1702 (59.3)	1	2053 (61.0)	1
Current	112 (93.3)	1.34 [0.64, 2.79]	121 (90.3)	0.82 [0.46, 1.49]	100 (64.9)	1.32 [0.94, 1.86]	79 (70.5)	1.59 [1.05, 2.40]
Past infection	44 (88.0)	0.65 [0.27, 1.55]	67 (91.8)	1.06 [0.45, 2.46]	44 (74.6)	2.04 [1.12, 3.69]	41 (62.1)	1.09 [0.66, 1.81]

1 Among the last 3 reported partners.

2 Current syphilis defined as TPPA positive and RPR positive. Past infection defined as TPPA positive and RPR negative.

**Table 4:** Factors independently associated with acceptance of VCT using the opt-out strategy (Team 1).

	Females (N = 2541)		Males (N = 3397)	
	number accepting (%)	Adjusted OR^1^ (95% CI)	number accepting (%)	Adjusted OR^2^ (95% CI)
**Education**		P = 0.34		**P = 0.05**
Secondary or higher	246 (89.1)	1	296 (86.6)	**1**
Primary	1976 (90.9)	1.17 [0.76, 1.82]	2737 (91.5)	**1.55 [1.10, 2.19]**
Incomplete primary	82 (94.3)	2.02 [0.74, 5.48]	54 (93.1)	**1.77 [0.61, 5.15]**
**Knowledge on HIV acquisition**		**P = 0.09**		P = 0.77
0–2 correct	858 (92.7)	**1**	132 (92.3)	1
3 correct	1444 (89.8)	**0.77 [0.57, 1.05]**	2137 (90.7)	0.96 [0.74, 1.25]
**Lifetime number of partners**		P = 0.58		**P = 0.03**
0	179 (92.8)	1.49 [0.80, 2.77]	292 (90.4)	**0.97 [0.58, 1.60]**
1	720 (90.3)	1	357 (90.6)	**1**
2 (F) or 2–4 (M)	659 (90.5)	1.02 [0.71, 1.46]	1231 (89.5)	**0.84 [0.57, 1.23]**
≥3 (F) or ≥5 (M)	742 (91.2)	1.12 [0.78, 1.62]	1204 (92.9)	**1.27 [0.84, 1.90]**
**Casual partner in past 12 m**		**P = 0.06**		P = 0.26
No	1981 (90.5)	**1**	1714 (90.2)	1
Yes	327 (93.2)	**1.52 [0.96, 2.38]**	1377 (92.1)	1.17 [0.89, 1.53]
**HSV-2**		**P = 0.04**		P = 0.22
Negative	1373 (92.1)	**1**	2258 (91.5)	1
Positive	920 (89.9)	**0.74 [0.56, 0.98]**	828 (91.0)	0.86 [0.66, 1.14]

1 Adjusted for all factors associated (p<0.10) with VCT uptake in females (knowledge on HIV acquisition, casual partner in past 12 m, and HSV-2 serostatus, shown in bold).

2 Adjusted for all factors associated (p<0.10) with VCT uptake in males (education and lifetime partners, shown in bold).

In males, in the unadjusted analysis, factors associated with VCT uptake using the opt-out strategy were lower education, high lifetime number of partners, and a concurrent sexual partnership or casual partner in the past 12 months (Table 3). In the multivariable analysis, only lifetime number of partners remained independently associated with VCT uptake (aOR = 1.27, CI = 0.84–1.90 for 5 or more partners vs one; Table 4). There was some evidence that VCT uptake was higher among those with lower education (aOR = 1.55, CI = 1.10–2.19 for primary vs secondary or above).

In both males and females, there was no evidence of an association of an individual's HIV serostatus with VCT uptake using the opt-out strategy (Table 3).

### Factors associated with VCT uptake in the opt-in strategy (Team 2)

In females, in the unadjusted analysis, factors associated (p<0.10) with VCT uptake using the opt-in strategy were non-Sukuma ethnic group, religion (uptake highest among Christians and lowest among those reporting traditional or no religion), knowledge of pregnancy prevention, and past or current syphilis infection (Table 3). In the multivariable analysis, factors that remained independently associated with VCT uptake at p<0.05 were religion, increasing knowledge of pregnancy prevention (aOR = 1.29, CI = 1.10–1.51, comparing 3 vs 0–2 correct answers), and current or past syphilis infection (aOR = 2.06, CI = 1.14–3.75 for past infection vs negative; Table 5). There was weak evidence that uptake was higher among non-Sukuma, after adjusting for other factors.

**Table 5:** Factors independently associated with uptake of VCT using the opt-in strategy (Team 2).

	Females (N = 3087)		Males (N = 3565)	
	number accepting (%)	Adjusted OR (95% CI)^1^	number accepting (%)	Adjusted OR (95% CI)^2^
**Currently married**		*P = 0.58*		**P = 0.004**
No	*809 (60.6)*	*1*	1486 (59.8)	**1**
Yes	*1038 (59.2)*	*0.96 [0.82, 1.11]*	688 (63.7)	**1.25 [1.07, 1.46]**
**Tribe**		**P = 0.09**		*P = 0.91*
Sukuma	1598 (59.3)	**1**	1893 (61.4)	*1*
Non-Sukuma	249 (64.0)	**1.24 [0.96,1.60]**	279 (58.4)	*0.99 [0.78, 1.25]*
**Religion**		**P = 0.004**		**P = 0.003**
Christian	1687 (60.9)	**1**	1819 (60.9)	**1**
Muslim	53 (54.1)	**0.77 [0.51, 1.16]**	59 (54.1)	**0.70 [0.47, 1.03]**
None/other	103 (48.1)	**0.62 [0.46, 0.83]**	252 (55.4)	**0.73 [0.59, 0.90]**
**Circumcised (males only)**				**P = 0.003**
Yes			782 (57.4)	**1**
No			1381 (63.4)	**1.28 [1.09, 1.51]**
**Knowledge on pregnancy prevention**		**P = 0.002**		*P = 0.33*
0–2 correct	564 (56.1)	**1**	524 (59.3)	*1*
3 correct	1277 (61.7)	**1.29 [1.10, 1.51]**	1644 (61.5)	*1.08 [0.92, 1.28]*
**Knowledge on STI acquisition**		*P = 0.35*		**P = 0.007**
0–2 correct	1208 (60.2)	*1*	1088 (59.4)	**1**
3 correct	633 (59.4)	*0.93 [0.79,1.09]*	1081 (62.8)	**1.22 [1.05, 1.40]**
**Slept away in past 12 m**		*P = 0.81*		**P = 0.003**
No	979 (59.0)	*1*	626 (56.5)	**1**
Yes	867 (60.9)	*1.02 [0.88,1.19]*	1546 (63.1)	**1.26 [1.08, 1.47]**
**Casual partner in past 12 m**		*P = 0.24*		**P = 0.002**
No	1581 (59.3)	*1*	931 (57.9)	**1**
Yes	266 (63.2)	*1.14 [0.92, 1.41]*	1243 (63.6)	**1.25 [1.09, 1.44]**
**Syphilis**		**P = 0.01**		*P = 0.23*
Negative	1702 (59.3)	**1**	2053 (61.0)	*1*
Current infection	100 (64.9)	**1.32 [0.94, 1.87]**	79 (70.5)	*1.43 [0.94, 2.18]*
Past infection	44 (74.6)	**2.06 [1.14, 3.75]**	41 (62.1)	*1.08 [0.65, 1.82]*

1 Adjusted for all factors associated (p<0.10) with VCT uptake in females (tribe, religion, knowledge of pregnancy prevention, and syphilis infection).

2 Adjusted for all factors associated (p<0.10) with VCT uptake in males (currently married, religion, circumcision, knowledge of STI acquisition, getting a girl pregnant in primary school, travel away in the past year, casual sexual partnership in the past year, and active syphilis.

In males, in the unadjusted analysis, factors associated with VCT uptake using the opt-in strategy were being married, religion (uptake highest among Christians, and lowest among Muslims), being uncircumcised, increased knowledge of HIV or STI acquisition, increasing number of lifetime partners, having made someone pregnant whilst still in primary school, travel outside the community in the past 12 months, reporting a concurrent sexual partnership or casual partner in the past 12 months, and current syphilis infection (Table 3). In the multivariable analysis, factors that remained independently associated with VCT uptake were being married (aOR = 1.25, CI = 1.07–1.46), religion, being uncircumcised (aOR = 1.28,–1.51), increasing knowledge of STI acquisition (aOR = 1.22, CI = 1.05–1.40, comparing 3 vs 0–2 correct answers), travel in the past 12 months (aOR = 1.26, CI = 1.08–1.47) and a casual sexual partner in the past 12 months (aOR = 1.25, CI = 1.09–1.44; Table 5).

In both males and females, there was no evidence of an association of an individual's HIV serostatus with VCT uptake using the opt-in strategy (Table 3).

## Discussion

We found that VCT uptake was significantly higher in the communities offered the opt-out strategy than the opt-in approach, in both males and females and in all age groups. The opt-out strategy was associated with a 50% increase in the overall prevalence of testing compared with the opt-in. Acceptance with the opt-out strategy was extremely high (90%), with no evidence of a difference by sex, age, or sociodemographic characteristics other than education level in males. In contrast, VCT uptake with the opt-in strategy varied among ethnic, religious or marital status groups.

Our results are consistent with other studies in sub-Saharan Africa, which show VCT uptake to be much higher with opt-out approaches than with client-initiated models [1], [13], [14], [15]. Although both VCT strategies in our study were researcher-initiated within a community-based trial, and so are not the same as VCT provision in non-research settings, some parallels can still be drawn. With our opt-in strategy, participants needed to make an active decision to seek out the VCT counsellor, with the chance that they might be seen entering the counsellor's room. With the opt-out strategy, the potential for perceived stigma was likely to have been reduced, since all participants were asked to visit the counsellor as part of the study procedures. The Regai Dzive Shiri (RDZ) trial of a multi-component adolescent HIV prevention intervention in Zimbabwe found that young people expressed a reluctance to test at a clinic because they were concerned it would suggest to their parents that they were sexually active [16]. Similar concerns could explain the lower uptake with the opt-in strategy in our setting. Alternatively, participants may have been anxious to get back to their daily activities, so chose not to prolong the study procedures by visiting the counsellor, indicating that VCT was not a high priority for them.

Despite this, we found that overall uptake of VCT, at 75%, was much higher than has been reported by other studies in this age group. In the RDZ trial, among those aged 18–24 years, uptake of VCT offered immediately after survey participation was 27% [16]. In Tanzania, a multi-site community randomised trial of a multi-component HIV prevention intervention including mobile VCT services, reported testing uptake of 30% among persons aged 18–32 years [17]. The extremely high uptake we found with the opt-out approach is similar to that from studies of home-based VCT delivery strategies that included adults of all ages [18], [19], [20], [21]. For example, a trial of home-based versus clinic-based HIV services in Uganda found that 89% of household members who were present at the time of the home visit accepted VCT [22].

We found no evidence of an association of VCT uptake with HIV serostatus, with either delivery strategy. Other studies have found an association between HIV status and testing strategy. In the Uganda trial, HIV prevalence among those testing in clinics was more than double that of those testing at home, suggesting that participants who tested in clinics may have suspected that they were HIV positive [22]. Similarly, in the RDZ trial, HIV prevalence among those testing in clinics was nearly double that in a researcher-initiated community setting [16]. However, since both our VCT approaches were researcher-initiated with very high uptake, our power to detect a difference in uptake by HIV status was limited.

Within each VCT strategy, males were as likely to accept testing as females. Although population-based surveys in many sub-Saharan African countries show that fewer men than women report ever having been tested for HIV, this may partly reflect opportunities for women to test during pregnancy [4]. Studies of research-initiated HIV testing do not suggest a consistent gender gap in uptake. A randomised controlled trial in Zimbabwe comparing workplace-based versus clinic-based VCT found no differences in uptake between men and women within each arm [23]. In the RDZ trial, although VCT uptake was lower among males than females in the clinic, there was no difference in the non-clinic based setting [16]. In a community-based survey in Kisesa, Tanzania, VCT uptake in village-based, research-initiated facilities was similar among males and females aged 15–24 years (12 vs 11%) [24].

Interestingly, we found different factors associated with VCT uptake within each strategy. With the opt-in approach, uptake varied by socio-demographic characteristics. Among both males and females, uptake was lower in those practising traditional or no religion, and in Muslims, compared with Christians. In females, uptake was slightly higher among non-Sukuma, and in males it was higher among those who were married. In contrast, VCT uptake under the opt-out strategy did not vary by these socio-demographic characteristics. In the community-based survey in Kisesa, Tanzania, uptake varied by ethnic group, and was lower among individuals practising traditional religion [24].

With our opt-out approach, there was some evidence that uptake was higher among females with lower knowledge of HIV acquisition and males with lower education. This could indicate lack of empowerment to decline VCT among those less educated, or greater fear of stigma and reluctance to test among those with more education. In contrast, in the opt-in strategy, uptake was associated with increased knowledge of pregnancy prevention (females) or STI acquisition (males), indicating that those with higher knowledge may have been more empowered to choose VCT. Hence, the higher level of self-efficacy resulting from better education/knowledge may have a different impact on uptake, depending on whether young persons need to actively choose (opt in) or refuse (opt out) testing.

In both strategies, there was some evidence that testing uptake was higher among participants reporting risky sexual behaviour. With the opt-out approach, females reporting a casual partner and males with ≥5 lifetime partners were somewhat more likely to test. With the opt-in approach, females with past or active syphilis, and males with a casual partner were more likely to test. These findings are similar to those from Kisesa, where VCT uptake among both males and females was associated with reporting a high-risk partner in the past 12 months [24].

There are limitations to our study. Since communities were not randomised to delivery strategy, there were imbalances in some sociodemographic characteristics between the two groups, in particular ethnic composition and education, which may have affected uptake and biased our estimate of the impact of the opt-out strategy. However, in the analysis adjusted for factors that were imbalanced, our estimate of the effect of strategy on uptake was almost unchanged, suggesting that there was no important confounding by these factors. VCT uptake in our research setting is likely to be context-specific, and the extent to which it can be generalised beyond this setting is uncertain. When consenting to the cross-sectional study, all participants agreed to provide a serum sample for anonymous HIV testing (a primary trial outcome). Thus, they may be different from young people in general who might be more reluctant to have their HIV results known, even if anonymised. Lastly, we did not collect data on the participants' perceptions of the acceptability of either VCT approach, so we cannot evaluate our results in the light of the views of the participants. Despite these limitations, our results demonstrate that a very high uptake of VCT among young persons in rural communities in Tanzania is feasible and acceptable.

Lastly, our findings have implications for universal voluntary testing programmes with immediate treatment (test-and-treat) for the prevention of HIV transmission. The barriers to testing that we identified when using the opt-in approach were no longer apparent when we used an opt-out approach, resulting in a consistently high uptake across the community.

In conclusion, VCT uptake among young people was extremely high when offered within a community-based opt-out strategy in this research context. Sociodemographic and behavioural factors affected uptake in different ways depending on the delivery strategy. Alternative approaches to increase the uptake of VCT among young persons are needed; our results indicate that opt-out strategies may contribute towards this goal.

## References

[1] Creek TL, Ntumy R, Seipone K, Smith M, Mogodi M, et al (2007) Successful introduction of routine opt-out HIV testing in antenatal care in Botswana. J Acquir Immune Defic Syndr 45: 102–107. 17460473 10.1097/QAI.0b013e318047df88

[2] Nakanjako D, Kamya M, Daniel K, Mayanja-Kizza H, Freers J, et al (2007) Acceptance of routine testing for HIV among adult patients at the medical emergency unit at a national referral hospital in Kampala, Uganda. AIDS Behav 11: 753–758. 17096199 10.1007/s10461-006-9180-9

[3] Kalichman SC, Simbayi LC (2003) HIV testing attitudes, AIDS stigma, and voluntary HIV counselling and testing in a black township in Cape Town, South Africa. Sex Transm Infect 79: 442–447. 14663117 10.1136/sti.79.6.442 PMC1744787

[4] WHO UNAIDS, UNICEF (2010) Towards universal access: scaling up priority HIV/AIDS interventions in the health sector. Progress Report.

[5] National Bureau of Statistics (NBS) Tanzania and ICF Macro (2011) Tanzania demographic and health survey 2010. Dar es Salaam, Tanzania: NBS and ICF Macro.

[6] WHO UNAIDS, UNICEF (2011) Global HIV/AIDS response: epidemic update and health sector progress towards universal access. Progress Report.

[7] WHO (2007) Access to health services for young people for preventing HIV and improving sexual and reproductive health. Department of Child and Adolescent Health and Development (CAH) Available: http://www.who.int/maternal_child_adolescent/data/media/adolescent_health_service_indicators_all.pdf.

[8] Ross DA, Changalucha J, Obasi AI, Todd J, Plummer ML, et al (2007) Biological and behavioural impact of an adolescent sexual health intervention in Tanzania: a community-randomized trial. AIDS 21: 1943–1955. 17721102 10.1097/QAD.0b013e3282ed3cf5

[9] Doyle AM, Ross DA, Maganja K, Baisley K, Masesa C, et al (2010) Long-term biological and behavioural impact of an adolescent sexual health intervention in Tanzania: follow-up survey of the community-based MEMA kwa Vijana trial. PLoS Medicine 7: e1000287. 20543994 10.1371/journal.pmed.1000287 PMC2882431

[10] Hayes RJ, Changalucha J, Ross DA, Gavyole A, Todd J, et al (2005) The MEMA kwa Vijana project: design of a community randomised trial of an innovative adolescent sexual health intervention in rural Tanzania. Contemp Clin Trials 26: 430–442. 15951245 10.1016/j.cct.2005.04.006

[11] Obasi AI, Cleophas B, Ross DA, Chima KL, Mmassy G, et al (2006) Rationale and design of the MEMA kwa Vijana adolescent sexual and reproductive health intervention in Mwanza Region, Tanzania. AIDS Care 18: 311–322. 16809108 10.1080/09540120500161983

[12] Hayes RJ, Moulton LH (2009) Cluster Randomised Trials. Boca Raton, Florida: Chapman and Hall/CRC 315.

[13] Bassett IV, Giddy J, Nkera J, Wang B, Losina E, et al (2007) Routine voluntary HIV testing in Durban, South Africa: the experience from an outpatient department. J Acquir Immune Defic Syndr 46: 181–186. 17667332 10.1097/QAI.0b013e31814277c8 PMC2140230

[14] Leon N, Naidoo P, Mathews C, Lewin S, Lombard C (2010) The impact of provider-initiated (opt-out) HIV testing and counseling of patients with sexually transmitted infection in Cape Town, South Africa: a controlled trial. Implement Sci 5: 8. 20205841 10.1186/1748-5908-5-8 PMC2825497

[15] Pope DS, Deluca AN, Kali P, Hausler H, Sheard C, et al (2008) A cluster-randomized trial of provider-initiated (opt-out) HIV counseling and testing of tuberculosis patients in South Africa. J Acquir Immune Defic Syndr 48: 190–195. 18520677 10.1097/QAI.0b013e3181775926 PMC2632747

[16] Chirawu P, Langhaug L, Mavhu W, Pascoe S, Dirawo J, et al (2010) Acceptability and challenges of implementing voluntary counselling and testing (VCT) in rural Zimbabwe: evidence from the Regai Dzive Shiri Project. AIDS Care 22: 81–88. 20390484 10.1080/09540120903012577

[17] Khumalo-Sakutukwa G, Morin SF, Fritz K, Charlebois ED, van Rooyen H, et al (2008) Project Accept (HPTN 043): a community-based intervention to reduce HIV incidence in populations at risk for HIV in sub-Saharan Africa and Thailand. J Acquir Immune Defic Syndr 49: 422–431. 18931624 10.1097/QAI.0b013e31818a6cb5 PMC2664736

[18] Were W, Mermin J, Bunnell R, Ekwaru JP, Kaharuza F (2003) Home-based model for HIV voluntary counselling and testing. Lancet 361: 1569. 10.1016/S0140-6736(03)13212-6 12737909

[19] Matovu JK, Makumbi FE (2007) Expanding access to voluntary HIV counselling and testing in sub-Saharan Africa: alternative approaches for improving uptake, 2001–2007. Trop Med Int Health 12: 1315–1322. 17949401 10.1111/j.1365-3156.2007.01923.x

[20] Mbopi-Keou FX, Ongolo-Zogo P, Angwafo F, Ndumbe PM, Belec L (2007) High impact of mobile units for mass HIV testing in Africa. Aids 21: 1994–1996. 17721120 10.1097/QAD.0b013e3282f006c3

[21] Fylkesnes K, Siziya S (2004) A randomized trial on acceptability of voluntary HIV counselling and testing. Trop Med Int Health 9: 566–572. 15117300 10.1111/j.1365-3156.2004.01231.x

[22] Lugada E, Levin J, Abang B, Mermin J, Mugalanzi E, et al (2010) Comparison of home and clinic-based HIV testing among household members of persons taking antiretroviral therapy in Uganda: results from a randomized trial. J Acquir Immune Defic Syndr 55: 245–252. 20714273 10.1097/QAI.0b013e3181e9e069

[23] Corbett EL, Dauya E, Matambo R, Cheung YB, Makamure B, et al (2006) Uptake of workplace HIV counselling and testing: a cluster-randomised trial in Zimbabwe. PLoS Med 3: e238. 16796402 10.1371/journal.pmed.0030238 PMC1483908

[24] Wringe A, Isingo R, Urassa M, Maiseli G, Manyalla R, et al (2008) Uptake of HIV voluntary counselling and testing services in rural Tanzania: implications for effective HIV prevention and equitable access to treatment. Trop Med Int Health 13: 319–327. 18397395 10.1111/j.1365-3156.2008.02005.x

